# Supplementary material for: Genomic prediction of stalk lodging resistance and the associated intermediate phenotypes in maize using whole‐genome resequence and multi‐environmental data
Source: Plant Genome. 2025 Sep 23;18(3):e70125. doi: 10.1002/tpg2.70125 (PMC12456121; doi:10.1002/tpg2.70125)
Supplement: Supplementary file 1 — Supplementary Table 1: Mean predictive abilities (PA) of MTM2 model considering CV1 and CV2. Supplementary Table 2: Mean predictive abilities (PA) of MTM2 model considering CV1 and CV2. Supplementary Figure 1: Comparison of predictive abilities between the original and LD‐pruned datasets based on the best‐performing model (MTM2) [file TPG2-18-e70125-s001.pdf]

Supplementary Table 1: Mean predictive abilities (PA) of MTM2 model considering CV1 and CV2. P-value was obtained from the paired one-sided Wilcoxon signed-rank test comparing to the single-environment GBLUP.

Trait	Environment	CV1		CV2	
		Mean PA	P-value	Mean PA	P-value
Bending strength	C20	0.34	0.14	0.56	$< 2.20 \times 10^{-16}$
	C21	0.43	0.32	0.69	$< 2.20 \times 10^{-16}$
	K20	0.38	0.02	0.89	$< 2.20 \times 10^{-16}$
	K21	0.36	0.02	0.64	$< 2.20 \times 10^{-16}$
Ear height	C20	0.20	0.3	0.78	$< 2.20 \times 10^{-16}$
	C21	0.21	2.69×10^{-3}	0.81	$< 2.20 \times 10^{-16}$
	K20	0.35	0.25	0.94	$< 2.20 \times 10^{-16}$
	K21	0.37	0.36	0.81	$< 2.20 \times 10^{-16}$
Flexural stiffness	C20	0.32	0.62	0.60	$< 2.20 \times 10^{-16}$
	C21	0.39	0.13	0.69	$< 2.20 \times 10^{-16}$
	K20	0.30	3.47×10^{-4}	0.88	$< 2.20 \times 10^{-16}$
	K21	0.29	0.11	0.62	$< 2.20 \times 10^{-16}$
Major diameter BI	C20	0.30	1.19×10^{-4}	0.62	$< 2.20 \times 10^{-16}$
	C21	0.29	0.02	0.62	$< 2.20 \times 10^{-16}$
	K20	0.28	0.19	0.89	$< 2.20 \times 10^{-16}$
	K21	0.36	0.06	0.63	$< 2.20 \times 10^{-16}$
Major diameter EI	C20	0.33	0.18	0.68	$< 2.20 \times 10^{-16}$
	C21	0.32	0.03	0.71	$< 2.20 \times 10^{-16}$
	K20	0.33	3.95×10^{-3}	0.92	$< 2.20 \times 10^{-16}$

	K21	0.33	1.10×10^{-2}	0.65	$< 2.20 \times 10^{-16}$
Minor diameter BI	C20	0.36	0.08	0.55	$< 2.20 \times 10^{-16}$
	C21	0.32	0.31	0.57	$< 2.20 \times 10^{-16}$
	K20	0.20	0.04	0.87	$< 2.20 \times 10^{-16}$
	K21	0.33	0.54	0.58	$< 2.20 \times 10^{-16}$
Minor diameter EI	C20	0.29	0.49	0.65	$< 2.20 \times 10^{-16}$
	C21	0.30	0.36	0.67	$< 2.20 \times 10^{-16}$
	K20	0.22	0.09	0.88	$< 2.20 \times 10^{-16}$
	K21	0.33	0.30	0.62	$< 2.20 \times 10^{-16}$
Plant height	C20	0.11	0.48	0.75	$< 2.20 \times 10^{-16}$
	C21	0.19	0.93	0.74	$< 2.20 \times 10^{-16}$
	K20	0.26	0.72	0.93	$< 2.20 \times 10^{-16}$
	K21	0.21	0.55	0.72	$< 2.20 \times 10^{-16}$
Rind thickness BI	C20	0.38	0.13	0.41	3.93×10^{-4}
	C21	0.17	0.50	0.22	0.01
	K20	0.22	0.04	0.89	$< 2.20 \times 10^{-16}$
	K21	0.23	0.16	0.30	8.55×10^{-6}
Rind thickness EI	C20	0.31	0.78	0.40	3.39×10^{-8}
	C21	0.17	5.61×10^{-3}	0.31	3.10×10^{-16}
	K20	0.22	0.31	0.88	$< 2.20 \times 10^{-16}$
	K21	0.26	0.28	0.32	6.48×10^{-8}

Supplementary Table 2: Mean predictive abilities (PA) of MTM2 model considering CV1 and CV2. P-value was obtained from the paired one-sided t-test comparing to the single-environment GBLUP.

Trait	Environment	CV1		CV2	
		Mean PA	p-value	Mean PA	p-value
Bending strength	C20	0.34	0.14	0.56	$< 2.20 \times 10^{-16}$
	C21	0.43	0.28	0.69	$< 2.20 \times 10^{-16}$
	K20	0.38	0.03	0.89	$< 2.20 \times 10^{-16}$
	K21	0.36	0.01	0.64	$< 2.20 \times 10^{-16}$
Ear height	C20	0.20	0.38	0.78	$< 2.20 \times 10^{-16}$
	C21	0.21	6.49×10^{-4}	0.81	$< 2.20 \times 10^{-16}$
	K20	0.35	0.32	0.94	$< 2.20 \times 10^{-16}$
	K21	0.37	0.39	0.81	$< 2.20 \times 10^{-16}$
Flexural stiffness	C20	0.32	0.62	0.60	$< 2.20 \times 10^{-16}$
	C21	0.39	0.21	0.69	$< 2.20 \times 10^{-16}$
	K20	0.30	3.54×10^{-4}	0.88	$< 2.20 \times 10^{-16}$
	K21	0.29	0.14	0.62	$< 2.20 \times 10^{-16}$
Major diameter BI	C20	0.30	9.60×10^{-5}	0.62	$< 2.20 \times 10^{-16}$
	C21	0.29	0.03	0.62	$< 2.20 \times 10^{-16}$
	K20	0.28	0.1	0.89	$< 2.20 \times 10^{-16}$
	K21	0.36	0.04	0.63	$< 2.20 \times 10^{-16}$
Major diameter EI	C20	0.33	0.15	0.68	$< 2.20 \times 10^{-16}$
	C21	0.32	0.03	0.71	$< 2.20 \times 10^{-16}$
	K20	0.33	1.45×10^{-3}	0.92	$< 2.20 \times 10^{-16}$
	K21	0.33	3.24×10^{-4}	0.65	$< 2.20 \times 10^{-16}$
Minor diameter BI	C20	0.36	0.06	0.55	$< 2.20 \times 10^{-16}$

	C21	0.32	0.27	0.57	< 2.20 x 10^{-16}
	K20	0.20	0.04	0.87	< 2.20 x 10^{-16}
	K21	0.33	0.67	0.58	< 2.20 x 10^{-16}
Minor diameter EI	C20	0.29	0.59	0.65	< 2.20 x 10^{-16}
	C21	0.30	0.33	0.67	< 2.20 x 10^{-16}
	K20	0.22	0.1	0.88	< 2.20 x 10^{-16}
	K21	0.33	0.33	0.62	< 2.20 x 10^{-16}
Plant height	C20	0.11	0.45	0.75	< 2.20 x 10^{-16}
	C21	0.19	0.92	0.74	< 2.20 x 10^{-16}
	K20	0.26	0.73	0.93	< 2.20 x 10^{-16}
	K21	0.21	0.42	0.72	< 2.20 x 10^{-16}
Rind thickness BI	C20	0.38	0.11	0.41	6.11 x 10^{-4}
	C21	0.17	0.52	0.22	0.01
	K20	0.22	0.09	0.89	< 2.20 x 10^{-16}
	K21	0.23	0.14	0.30	2.15 x 10^{-6}
Rind thickness EI	C20	0.31	0.72	0.40	6.62 x 10^{-9}
	C21	0.17	0.01	0.31	< 2.20 x 10^{-16}
	K20	0.22	0.31	0.88	< 2.20 x 10^{-16}
	K21	0.26	0.18	0.32	2.16 x 10^{-8}

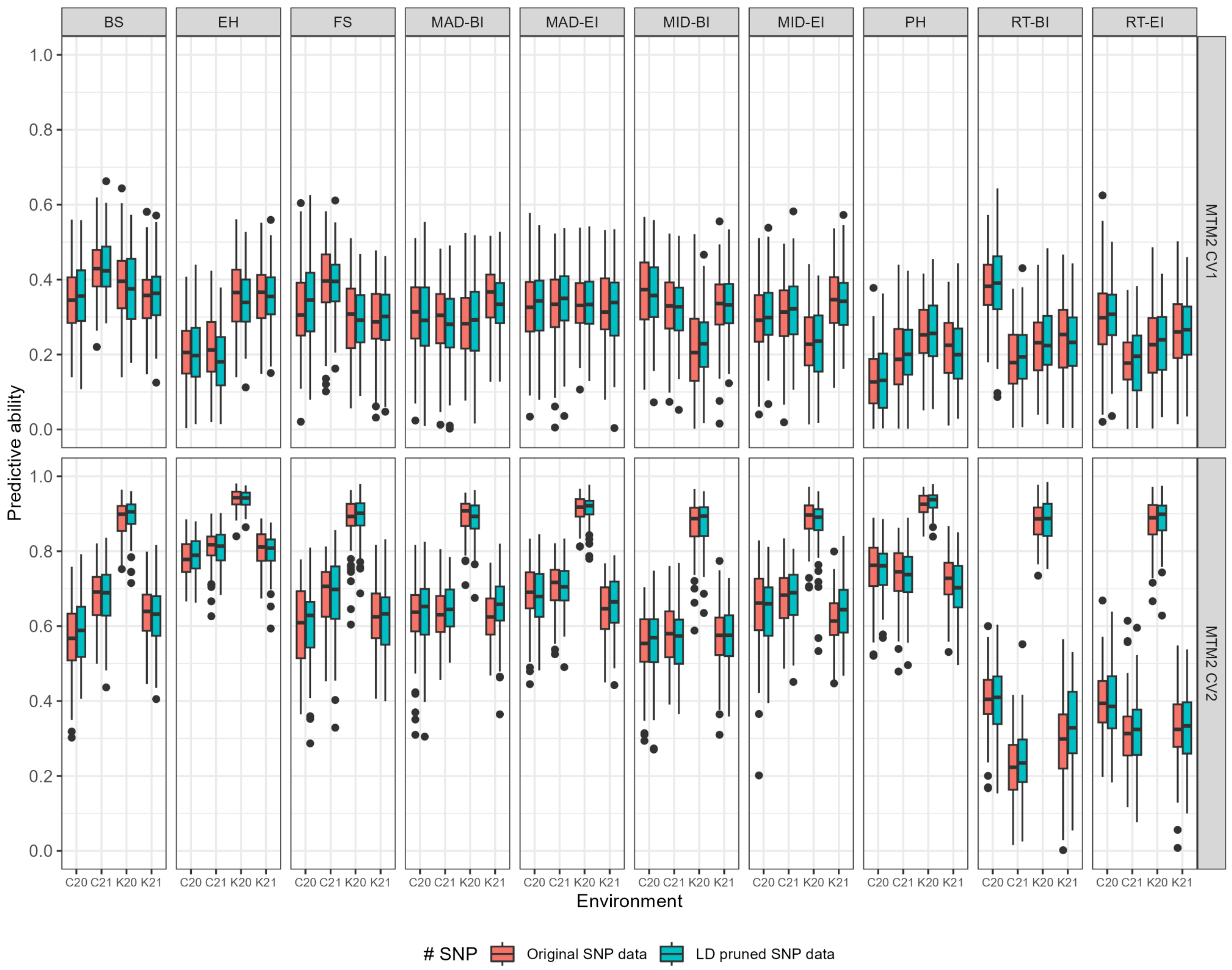

Supplementary Figure 1: Comparison of predictive abilities between the original and LD-pruned datasets based on the best-performing model (MTM2).
